# Influence of the Original Concrete Strength and Initial Moisture Condition on the Properties Improvement of Recycled Coarse Aggregate via Accelerated Carbonation Reactions

**DOI:** 10.3390/ma17030706

**Published:** 2024-02-01

**Authors:** Xueli Ju, Linjian Wu, Mingwei Liu, Han Jiang, Wenxiao Zhang, Li Guan, Xiang Chen, Xinhui Fan

**Affiliations:** 1 National Engineering Research Center for Inland Waterway Regulation, School of River and Ocean Engineering, Chongqing Jiaotong University, 66 Xuefu Road, Nan’an District, Chongqing 400074, China; juxueli97@126.com (X.J.); 15213118329@163.com (H.J.); zwx126737@126.com (W.Z.); 17830472747@126.com (X.C.); 18296296009@126.com (X.F.); 2 Sichuan Communication Surveying & Design Institute Co., Ltd., Taisheng Bei Road, Qingyang District, Chengdu 610017, China; guanli9798@126.com

**Keywords:** CO_2_ curing, recycled coarse aggregate, original concrete strength, initial moisture condition, properties improvement

## Abstract

The physical and mechanical properties of recycled coarse aggregate (RCA) are worse than those of natural coarse aggregate (NCA), and the overall performance of recycled concrete prepared from RCA is worse than that of natural aggregate concrete. Treatment of RCA by CO_2_-accelerated carbonation effectively improves the macroscopic properties of RCA. The degree of influence of raw material factors, i.e., the original concrete strength (OCS) and initial moisture content (IMC) of RCA, on the carbonation of RCAs is very complex. Herein, an accelerated carbonation experiment for RCA with different material factors as variables was carried out to explore the influence of the abovementioned factors on the physical properties of carbonated recycled coarse aggregate (CRCA). By analyzing the microstructure of the RCA with the best modification effect before and after carbonation, the carbonation modification mechanism of the RCA was revealed. The physical performance indexes, including the apparent density, water absorption and carbonation rate, of the dried RCA with an OCS of C40 and C50 were significantly improved. The research results can provide basic data and theoretical support for promoting the popularization and application of RCA and recycled concrete in practical engineering.

## 1. Introduction

Concrete is the most widely used building material in the construction industry and has the greatest impact on total emissions and natural resource consumption in the construction industry [1,2,3,4,5]. Therefore, the construction industry is striving to achieve sustainable development through technological innovation and the use of recycled materials to protect the environment and reduce dependence on natural resources [6,7,8]. For example, crushing concrete waste into recycled coarse aggregate (RCA), partially or completely replacing natural coarse aggregate (NCA) in concrete, is a leading choice for concrete waste recycling for scholars worldwide [9,10,11]. However, since the surface of RCA is covered with old mortar with a loose pore structure, and on account of the interfacial transition zone (ITZ) between old aggregate and mortar, its performance is lower than that of NCA [12,13,14], and its durability is worse, which seriously restricts the application of RCA in green port construction.

Based on the alkali aggregate characteristics of RCA, the macroscopic properties of RCA, including the apparent density, water absorption, actual mass increase and carbonation rate, can be effectively improved by using CO_2_-actived carbonation of RCA [15,16,17,18]. The reason is that cement-based materials can generate dense silica gels and CaCO_3_ through the carbonation reaction, which can densify the old mortar attached to RCA and the pores in the old ITZ, improve its microstructure, and improve the macroscopic properties of RCA [19,20,21]. According to previous research, the factors affecting RCA carbonation can be divided into two categories: carbonated environmental factors and raw material factors of RCA [15,22]. The carbonation environment factors that affect the performance improvement of RCA include CO_2_ concentration, temperature, pressure, relative humidity (RH), and carbonation time [15,23,24]. In addition, the RCA raw material factors mainly include the original concrete strength (OCS) of the RCA and the initial moisture content (IMC) of the RCA, etc. [25,26,27,28].

The raw material factors of RCA have a considerable effect on its physical and mechanical properties before and after carbonation [29,30]. Scholars have found that the OCS, as an index to comprehensively evaluate the original concrete performance, has a very significant impact on the macroscopic properties of RCA and carbonated recycled coarse aggregate (CRCA) [24,26]. The higher the OCS, the greater the number of hydration products of the RCA and the better the performance of CRCA [31]. However, this does not mean that the OCS is proportional to the carbonation effect. When the OCS is high, the adhesion density of RCA to the old mortar increases, and the permeability of CO_2_ to RCA decreases, which is not conducive to the improvement of RCA performance.

The IMC plays a dominant role in the carbonation efficiency and modification effect of RCA. The free water in the pores of RCA is the basis of the carbonation reaction. When the external CO_2_ diffuses into the pores, it dissolves into the water and carbonizes with the hydration products in the form of H_2_CO_3_. The CO_2_ diffusion rate in water is much lower than that in the atmosphere. An excessive water content will affect CO_2_ diffusion [32], but an insufficient water content will hinder CO_2_ dissolution, and water will also affect the precipitation of Ca^2+^ [33]. In other words, an IMC that is too high or too low will negatively affect the carbonation efficiency of RCA. Theoretically, there is an optimal IMC for the accelerated carbonation of RCA.

The carbonation modification of RCA by CO_2_ will densify the microstructure of RCA [34,35]. Pan et al. [26] found that the porosity of carbonated recycled fine aggregate decreased by 57.9% compared with that of uncarbonated recycled fine aggregate. Xuan et al. [36] found, via scanning electron microscopy, that carbonation treatment can eliminate the macropores of RCA above 200 nm and can effectively reduce the mesopores in the range of 50–200 nm. This is because the hydration products such as CH and C-S-H inside RCA are carbonated to form various forms of CaCO_3_, including fibrous, massive and plate-like forms, which can fully fill the cracks.

In summary, although there have been some studies on the use of CO_2_ to accelerate carbonation and improve the performance of RCA, in view of the fact that the influence of the OCS and IMC on the carbonation modification effect of RCA and RCAC is very complicated, the influence of the IMC on the performance of CRCA has rarely been reported in the literature. In this paper, an indoor accelerated carbonation experiment of RCAs with different raw material characteristics is carried out to explore the change in the physical performance index of RCAs with changes in the OCS and IMC before and after carbonation. The influence of the OCS and IMC on the carbonation modification effect of RCA is comprehensively analyzed, and the magnitudes of the OCS and IMC of RCA are determined when the carbonation modification effect is optimal. On this basis, a microstructure analysis of the RCA with the best carbonization modification effect before and after carbonization was carried out, and the mechanism of the influence of the carbonization reaction on the modification of RCA was analyzed in depth. The research results can provide basic data and theoretical support for promoting the popularization and application of RCA and recycled concrete in practical engineering.

## 2. Materials and Methods

### 2.1. Raw Materials and Mixture Proportions for the Original Concrete of RCA

PC.42.5R composite Portland cement with an apparent density of 3.1 × 10^3^ kg/m^3^, natural river sand (fine aggregate) with a fineness modulus of 2.58, ordinary tap water (mixing water) with a density of 1 × 10^3^ kg/m^3^, and continuous graded natural gravel (coarse aggregate) with a nominal particle size of 5–20 mm and an apparent density of 2.69 × 10^3^ kg/m^3^ were selected as the original concrete raw materials for preparing RCA in this paper [37].

According to the specifications [38], to ensure the durability of reinforced concrete in marine environments, it is necessary to use a minimum strength grade of C30. Previous studies have demonstrated that using recycled concrete aggregate with excessively high original concrete strength is not suitable for carbonation modification. Therefore, three RCAC strength grades, C30, C40, and C50, were designed in this study. To achieve the predetermined strength grade, referring to the specifications [39], the standard mix proportion of the original concrete under each strength grade was calculated, as shown in Table 1. The preparation and maintenance of the original concrete were carried out in accordance with the specifications.

### 2.2. RCA Production and Preparation

After pouring the original concrete into a plastic mold measuring 100 × 100 × 100 mm^3^, the specimens were transferred to a vibration table for compaction through vibration. For the curing process, the specimens were initially placed in a standard curing box at a temperature of 20 ± 5 °C and a relative humidity of 90% for 24 h. Following the initial curing, the concrete specimens were then immersed and cured in a saturated calcium hydroxide solution for 28 days. After the curing of the test specimens, according to the design strength category of the original concrete specimen, a jaw crusher was utilized for crushing. After the parent concrete specimen was crushed, the RCA was sieved according to the nominal particle size to obtain the RCA with a particle diameter range of 10–20 mm. The RCAs were pretreated to obtain three RCA samples under three water-bearing states:

(1) Complete drying state: the RCA sample was poured into a shallow plate and dried to a constant weight at 105 ± 5 °C in a blast oven.
(2) Untreated state: the RCA after crushing and screening was used directly for reserve, without any treatment.
(3) Complete wetting state: the RCA was soaked in water for 24 h for fully saturated water treatment and removed with a wet towel to dry the water on the surface of the sample to prepare a saturated surface dry sample for use.

**Figure 1:**
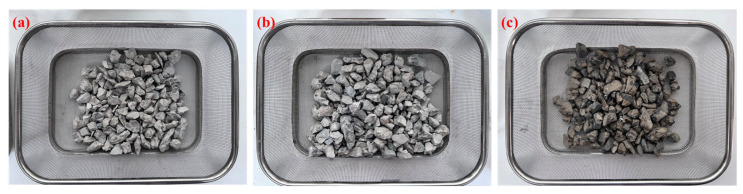
RCA with different IMCs: (**a**) complete drying; (**b**) untreated; (**c**) complete wetting.

As shown in Figure 1, in this experiment, RCAs with three OCSs (C30, C40, C50) and three different IMCs (complete drying, untreated, complete wetting) were designed for subsequent carbonation modification experimental research.

### 2.3. CO_2_ Curing Treatment Method for RCA

Based on the above design, RCAs with different OCSs and IMCs were obtained. An indoor accelerated carbonation experiment to determine the influence of different raw material factors on the physical properties of CRCAs was carried out. The experimental conditions are detailed in Table 2. Each group of RCA samples included 500 g tiles placed in a carbonation container and placed in a concrete carbonation box with an ambient temperature of 20 °C, a relative humidity of 70%, and a CO_2_ concentration of 20% for standard carbonation. To ensure the accuracy of the experimental results, three groups of parallel samples were considered under each working condition.

**Figure 2:**
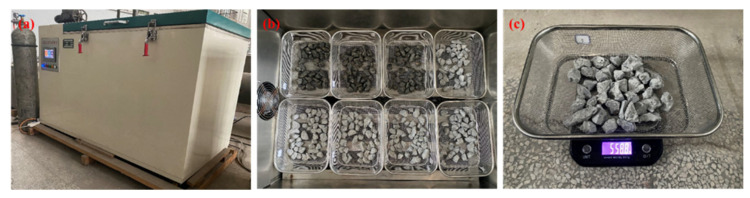
RCA carbonated and weighed: (**a**) concrete carbonation box; (**b**) RCA carbonated; (**c**) RCA weighed.

After the carbonation began, each sample was weighed and the quality was recorded at certain intervals until carbonation ended, at which point the sample quality remained basically unchanged. At this time, the RCA was considered to be completely carbonated. After the carbonation of the RCA, the complete CRCA samples were put into an oven at a temperature of 105 ± 5 °C to dry until a constant weight and the corresponding quality was recorded, as shown in Figure 2. Three parallel samples were mixed evenly to measure the physical performance index of CRCA.

### 2.4. Determination of the Properties of Coarse Aggregate

#### 2.4.1. Apparent Density

The apparent density is the ratio of material mass to the apparent volume. In this paper, the apparent density of aggregates was measured using the wide-mouth bottle method [40]. The apparent density is given by Equation (1):

(1) $$\rho_{a}=\left(\frac{m_{d}}{m_{d}+m_{o}-m_{t}}\right)\times \rho_{w}$$

where *ρ_a_* is the apparent density of the aggregate (kg/m^3^). *m_d_* is the mass of the aggregate after drying (g). *m_t_* is the total mass of the aggregate, water, jar and glass sheet (g). *m_o_* is the total mass of the aggregate, jar and glass sheet (g). *ρ_w_* is the density of water (1000 kg/m^3^).

#### 2.4.2. Water Absorption

According to the specification [40], the water absorption was calculated as shown in Equation (2):

(2) $$W_{a}=\frac{m_{s}-m_{d}}{m_{d}}\times 100\%$$

where *W_a_* is the water absorption rate of the aggregate (%). *m_s_* is the mass of the saturated surface dry aggregate (g). *m_d_* is the mass of aggregate after drying (g).

The apparent density and water absorption results of NCA and RCA are shown in Table 3.

#### 2.4.3. Moisture Content

The moisture contents of RCAs before and after carbonation modification were tested, according to the specification [40]. For the moisture content calculation expression, see Equation (3):

(3) $$\omega =\frac{m_{n}-m_{d}}{m_{d}}\times 100\%$$

where *ω* is the moisture content of the aggregate (%). *m_n_* is the mass of aggregate before drying (g). *m_d_* is the mass of aggregate after drying (g).

The moisture content test results of RCAs with different IMCs are shown in Table 4.

#### 2.4.4. Mass Variation and Carbonation Ratio of CRCA

By weighing the RCAs before and after carbonation to determine the increase in the quality of RCAs, Δ*M* was calculated as follows:

(4) $$\Delta M=M_{n}-M_{0}\cdot \left(1-\omega_{i}\right)$$

where Δ*M* denotes the mass increase of RCA (g). *M_n_* is the mass of the RCA at complete carbonation (g). *M*_0_ is the initial mass of RCA (g). *ω_i_* is the initial moisture content of RCA (%).

In this paper, RCAs with different IMCs were designed, and the RCA still contained a large amount of water after carbonation. It was necessary to further dry the RCAs to obtain the actual mass increase, Δ*M_e_*. The calculation was performed as follows:

(5) $$\Delta M_{e}=M_{H}-M_{0}\cdot \left(1-\omega_{i}\right)$$

where Δ*M_e_* is the actual mass increase of RCA (g). *M_H_* is the mass of the RCA after complete carbonation and drying (g).

To quantify the carbonation degree of RCAs, many scholars have carried out relevant research. At present, the empirical formula for calculating the carbonation rate of RCA is given by Equation (6):

(6) $$\varepsilon =\frac{\Delta M_{e}}{\Delta M_{t}}\times 100\%$$

where *ε* is the carbonation rate of the RCA (%). Δ*M_e_* is the actual mass increase of RCA (g). Δ*M_t_* is the maximum theoretical absorption of CO_2_ by cement mortar adhered to the surface of RCA, calculated as shown in [25]:

(7) $$\Delta M_{t}=\frac{M_{c}\cdot {\mathrm{CO}}_{2}{\%}_{\max}}{\left(M_{c}+M_{s}+M_{a}+0.23\cdot M_{c}\right)\left(1+\omega_{i}\right)}$$

where *M_c_*, *M_s_* and *M_a_* are the proportions (by mass) of cement, sand and coarse aggregate in the original concrete, respectively (%). CO_2_%_max_ is the maximum theoretical amount of CO_2_ captured by Portland cement. In this experiment, CO_2_%_max_ = 50% [25].

## 3. Results and Analysis

### 3.1. Macroscopic Properties of RCA before and after Carbonation

#### 3.1.1. Apparent Density

**Figure 3:**
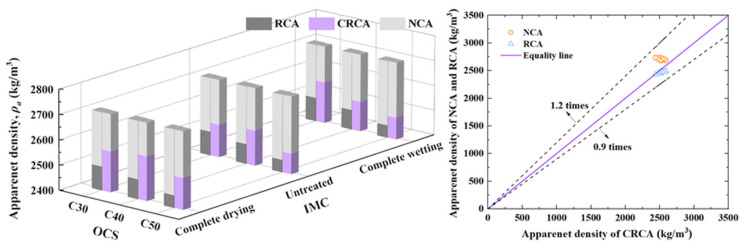
The measured values of *ρ_a_* of NCAs, RCAs and CRCAs.

Figure 3 shows the measured results of the apparent density of NCA, RCA and CRCA with different OCSs and IMCs. It can be seen from the figure that the apparent density of RCA and CRCA was smaller than that of NCA, and the apparent density of CRCA was 0.3–3.95% higher than that of RCA. This is because the carbonization product densified the pores of the RCA and improved the loose and porous morphology inside the aggregate.

To quantitatively describe the difference in apparent density among NCA, RCA and CRCA, the three were compared, and the results are shown in Figure 3. It can be seen that the apparent density of NCA was approximately 1.2 times that of CRCA, while the apparent density of RCA was approximately 90% that of CRCA.

To quantitatively evaluate the improvement in the apparent density of RCA via carbonation treatment, the apparent density increase rate ∆*ρ_a_* was defined as follows:

(8) $$\Delta \rho_{a}=\frac{\rho_{a\mathrm{CRCA}}-\rho_{a\mathrm{RCA}}}{\rho_{a\mathrm{RCA}}}\times 100\%$$

where *ρ_a_*_CRCA_ is the apparent density of CRCA (kg/m^3^). *ρ_a_*_RCA_ is the apparent density of RCA (kg/m^3^).

**Figure 4:**
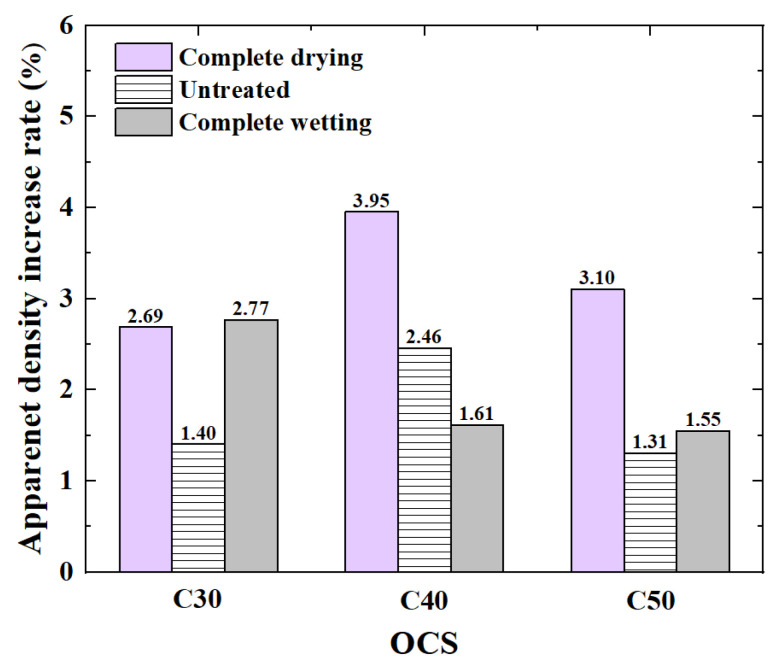
The variation in Δ*ρ_a_* of RCAs after carbonation.

The variation in Δ*ρ_a_* with changes in the OCS and IMC is shown in Figure 4.

(1) The influence of OCS on apparent density

It can be seen from Figure 3 that the apparent density of RCA and CRCA decreased with increasing the OCS at any IMC. This is because the RCA (CRCA) with a low grade of OCS had low compactness, greater pore distribution and a smaller apparent density. Quantitative analysis showed the following results (Figure 4):

In the complete drying condition, the Δ*ρ_a_* of the RCA with C40 reached a peak after carbonation, which was 3.95%, and this improvement was the most significant. According to the analysis, this was due to the low compactness of the RCA with a low OCS, a high pore distribution, a limited internal carbonizable material [15], while the C50 RCA with the highest OCS had a high compactness and more difficult CO_2_ penetration during the carbonation process. Therefore, the RCA with the medium OCS C40 could not only provide abundant carbonizable materials (CH, C-S-H), but also had sufficient carbonation space, and the modification effect was the best.

In the untreated condition, the Δ*ρ_a_* of the RCA after carbonation increased first and then decreased with increasing OCS. The Δ*ρ_a_* of the RCA with the medium OCS grade C40 after carbonation was the largest, and the improvement was remarkable.

Under complete wetting conditions, the Δ*ρ_a_* of the RCA after carbonation decreased with increasing OCS, and the Δ*ρ_a_* of the RCA with an OCS of C30 reached the peak value.

In summary, in this study, the Δ*ρ_a_* of RCAs with higher or lower OCSs after carbonation was not uniform with the change in the OCS, and the carbonation process was evidently limited by the IMC. In general, the improvement in the apparent density of RCA with C40 was the best.

(2) The influence of IMC on apparent density

It can be seen from Figure 4 that compared with those in the untreated condition and the complete wetting condition, the Δ*ρ_a_* of the dried RCA with an IMC of 0 after carbonation was greater, indicating that reducing the IMC of the RCA is an effective measure to increase the apparent density of the CRCA.

#### 3.1.2. Water Absorption

**Figure 5:**
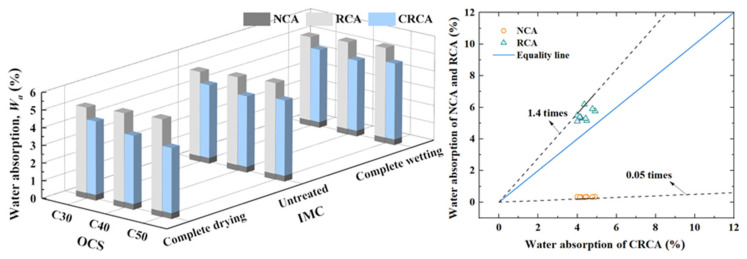
The measured values of *W_a_* of NCAs, RCAs and CRCAs.

Figure 5 shows the measured results of water absorption of NCAs, RCAs with different OCSs, and IMCs, as well as the result for CRCAs. It can be seen from the figure that the water absorption of the RCAs and CRCAs is much higher than that of NCAs, and the water absorption of CRCA was 7.98–29.56% lower than that of uncarbonated recycled aggregate. This is because the carbonation products densified the pores of the RCAs and improved the morphology of the loose and porous structure inside the aggregate, resulting in a decrease in their water absorption capacity.

To quantitatively describe the difference in water absorption of NCAs, RCAs and CRCAs, the three were compared, and the results are shown in Figure 5. It can be seen that the water absorption of the RCAs and CRCAs was approximately 20 times and 28 times that of the NCAs. The water absorption rate of the RCAs was approximately 1.4 times that of the CRCAs.

To quantitatively evaluate the degree of reduction in water absorption of RCAs via carbonation, the water absorption reduction rate Δ*W_a_* was defined as follows:

(9) $$\Delta W_{a}=\frac{W_{a\mathrm{CRCA}}-W_{a\mathrm{RCA}}}{W_{a\mathrm{RCA}}}\times 100\%$$

where *W_a_*_CRCA_ is the water absorption of CRCA (%). *W_a_*_RCA_ is the water absorption of RCA (%).

**Figure 6:**
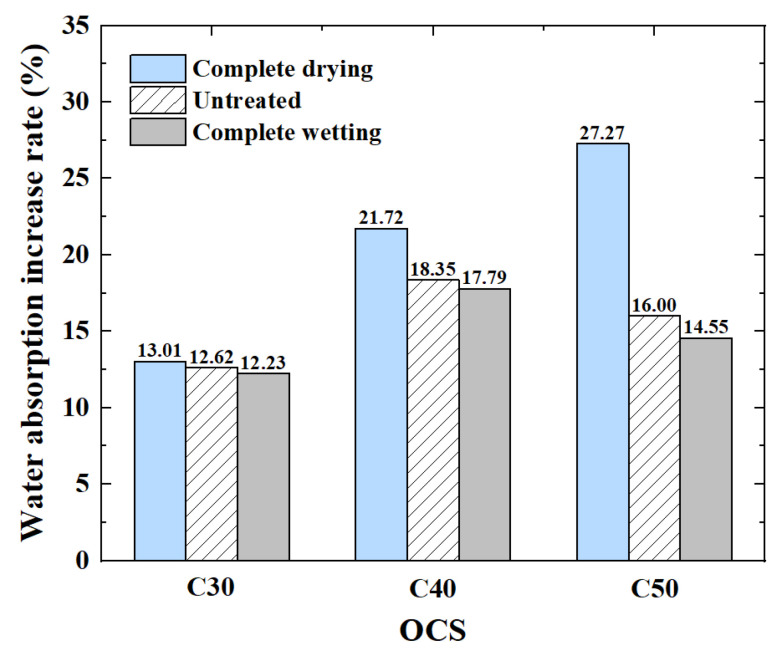
The variation in Δ*W_a_* of RCAs after carbonation.

The variation in Δ*W_a_* with the OCS and IMC is shown in Figure 6.

(1) The influence of OCS on water absorption

Figure 5 shows that the water absorption of the RCAs increased with increasing OCS. The water absorption of the CRCAs decreased almost linearly with increasing OCS. The quantitative analysis shows that (Figure 6) in the complete drying condition, the Δ*W_a_* of the RCAs increased with increasing OCS. The Δ*W_a_* of the RCA with an OCS of C50 decreased the most after carbonization, with a value of 29.56%, and the improvement was the most significant. In the untreated condition and complete wetting condition, the Δ*W_a_* of the RCA after carbonization decreased first and then increased with increasing OCS. The Δ*W_a_* of the RCA with OCS C50 after carbonization was the largest.

In summary, under complete drying conditions, the water absorption rate of the RCA with a higher OCS was the largest after carbonation; after the carbonation of the untreated and completely wetted RCAs, the degree of water absorption reduction was affected by the OCS.

(2) The influence of IMC on water absorption

Figure 6 shows that compared with the untreated and completely wetted RCAs, the dried RCA with an IMC of 0 had a significantly reduced water absorption after carbonation. Therefore, under the carbonation environment of an ambient temperature of 20 °C, a CO_2_ concentration of 20% and a relative humidity of 70%, reducing the IMC of the RCAs significantly reduced the water absorption of the CRCAs.

#### 3.1.3. Moisture Content

**Figure 7:**
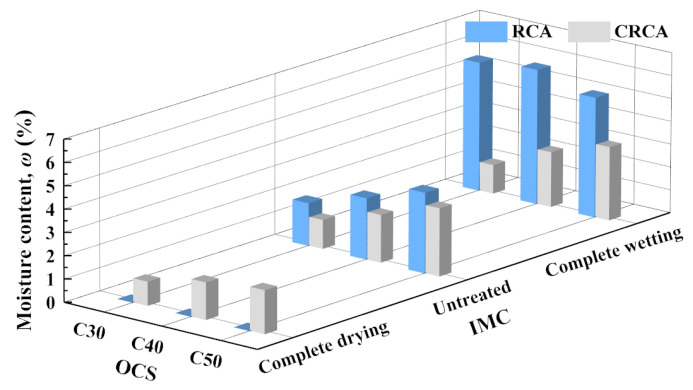
The measured values of moisture content of NCAs, RCAs and CRCAs.

Figure 7 shows the measured results of the moisture content of RCAs and CRCAs with different IMCs.

(1) The influence of OCS on moisture content

In the untreated condition, the moisture content of the RCAs increased almost linearly with increasing OCS. Under complete wetting conditions, the moisture content of the RCAs decreased with increasing OCS. Under any IMC condition, the water content of the CRCAs increased almost linearly with increasing OCS. This was due to the lower OCS of the RCA hydration products, which had a limited ability to absorb water in the carbonation process [15].

(2) The influence of IMC on moisture content

**Figure 8:**
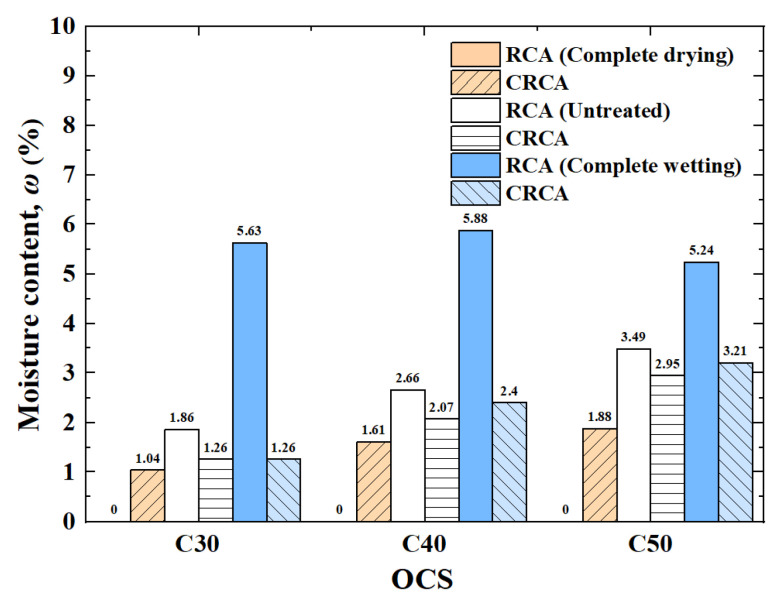
The measured values of moisture content of RCAs and CRCAs.

The moisture content of the dried RCA increased significantly after the carbonation treatment and absorption of water from the environment. After the carbonation of the untreated and completely wetted RCAs, the moisture content of the CRCAs decreased to varying degrees. Compared with that of the untreated RCAs, the moisture content of the completely wetted CRCAs decreased the most. It can be seen from Figure 8 that with the same OCS, the RCA with a lower IMC absorbed more water from the environment, and the moisture content after carbonation was higher.

#### 3.1.4. Mass Increase and Actual Mass Increase

(1) The influence of OCS on mass increase

**Figure 9:**
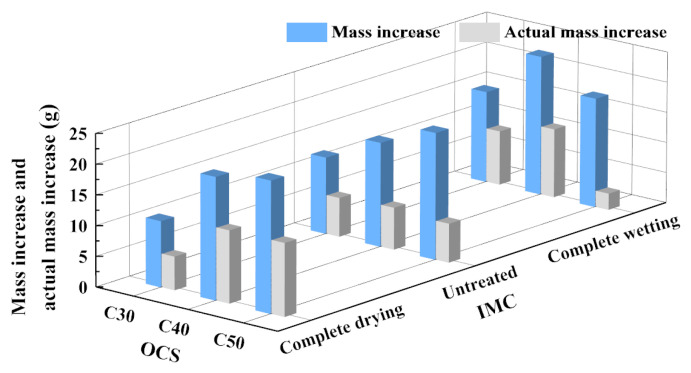
The mass increase and actual mass increase of RCAs before and after carbonation.

Figure 9 shows the results of the mass increase and the actual mass increase of the RCAs after carbonation. From Figure 9, it can be seen that the mass increase of the RCAs after carbonation increased with increasing OCS in the drying condition and untreated condition. In the complete wetting condition, the mass increase of the RCA increased first and then decreased with increasing OCS. In the complete drying condition, the actual mass increase of the RCA with the C50 OCS after complete carbonation reached a peak, with a value of 13.3 g. In the untreated condition and the complete wetting condition, the actual mass increase of the RCA with the C40 OCS after complete carbonation was larger.

(2) The influence of IMC on mass increase

It can be seen from Figure 9 that there was a close relationship between the IMC of the RCAs and the actual mass increase. The RCA with C30 had the largest actual mass increase after carbonation in the complete wetting condition, while the RCAs with C40 and C50 had the largest actual mass increase after carbonation in the complete drying condition. The actual mass increase of the CRCA with C50 was most significantly affected by the IMC. The lower the IMC, the greater the actual mass increase.

#### 3.1.5. Carbonation Ratio

**Figure 10:**
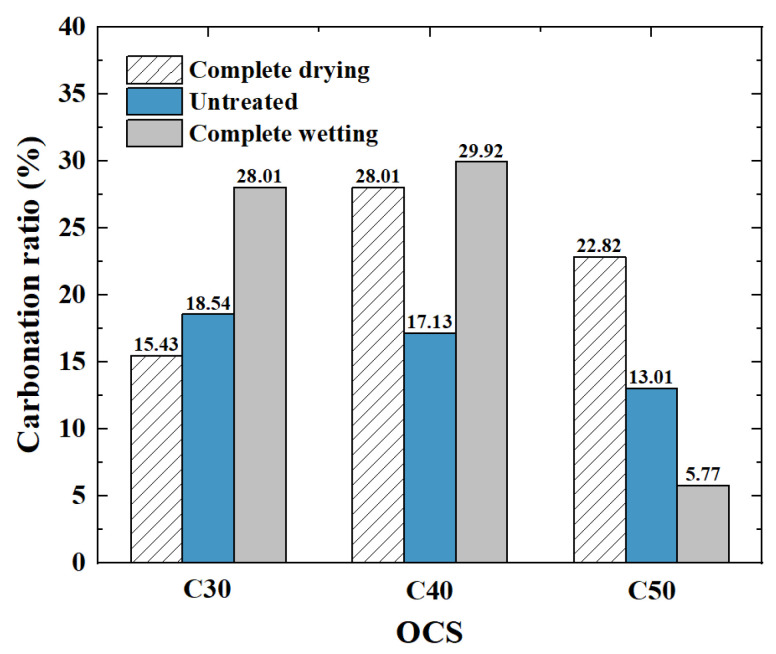
Carbonation rates of RCAs.

The actual mass increase, in all cases, was further calculated, and the variations in the carbonation rates of the RCAs with various OCSs for each IMC were obtained. As shown in Figure 10, the influence of the OCS on the carbonation ratio can be calculated.

(1) Influence of the OCS on the carbonation ratio

In the complete drying condition, the carbonation rate increased first and then decreased with the increasing OCS. The OCS increased from C30 to C40, and the carbonation rate increased by 81.5%. When the OCS increased from C40 to C50, the carbonation rate decreased by 18.5%. The carbonation rate of RCA with C40 was the highest, with a value of 28.1%. In the untreated condition, the carbonation rate decreased with an increase in OCS, but the degree of reduction was not significant (about 7.6–24.1%). In the complete wetting condition, the carbonation rate increased first and then decreased with the increasing OCS. The RCA carbonation rates corresponding to the OCSs of C30 and C40 reached their peaks, which were 28.01% and 29.92%, respectively. When the OCS was increased to C50, the carbonation rate decreased by about 80.7%. In general, the overall carbonation rate of the RCA with C40 was higher. The carbonation rate of the RCA under all cases of the OCS exceeded 15%, and the average carbonation rate reached 22.15%. The carbonation rate of the completely dried RCA with C40 was the highest, with a value of 28.1%. With an OCS of C30, the carbonation rate of the RCA exceeded 15% in seven groups, and the average carbonation rate was 19.20%. The lowest occurred for the RCA with C50. There were three groups of carbonation rates ≤10% with an OCS of C50, and the average carbonation rate was only 14.59%.

In summary, when the OCS was too high (C50) or too low (C30), the carbonation rate of the RCA was low, while the carbonation rate of the RCA with the medium OCS (C40) was the highest. This paper describes RCAs with different OCS grades. The carbonizable material and the overall compactness for RCAs with a low OCS grade was relatively loose. The hydration product was limited, its capture and absorption of CO_2_ were limited, and the overall carbonation rate was low. The RCA with a high OCS grade had more carbonation material attached to the hardened cement paste, but the overall density was larger, and the carbonation medium was difficult to penetrate, resulting in a more difficult carbonation reaction. This is consistent with the results in reference [31]; the RCA with the medium OCS grade was in a state of equilibrium.

(2) Influence of the IMC on the carbonation ratio

At a low OCS (C30), the carbonation rate of RCA increases with an increase in the IMC. In other words, in the complete wetting condition, the carbonation rate reaches its peak, which is 28.01%. The carbonation rate increases from the complete drying condition to the saturated state, and the carbonation rate increases by 81.5%. Under the C40 OCS, the carbonation rate of RCA increases first and then decreases with an increase in the IMC, and the RCA carbonation rates in the complete drying and complete wetting condition are very close to that in the untreated condition, increasing by about 74.7%. Under a high OCS (C50), the carbonation rate of RCA decreases with an increase in the IMC. In other words, under the complete drying condition, the carbonation rate reaches a peak of 22.82%. From the complete drying state to the saturated state, the carbonation rate is reduced by 74.7%. A high IMC is not conducive to the carbonation of RCA, which makes the carbonation rate of RCA worse than that of other initial moisture conditions [41].

In summary, increasing the IMC of the RCA and the difference in the internal and external humidity gradients in the carbonation environment can effectively improve the carbonation degree of the RCA.

### 3.2. Microanalysis of RCAs before and after Carbonation

**Figure 11:**
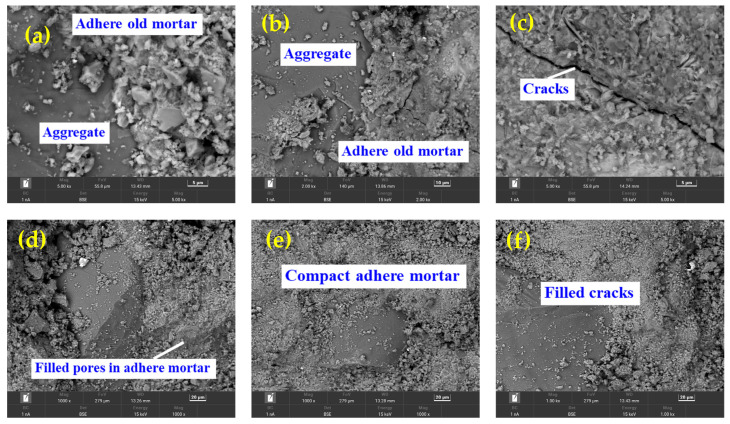
Microscopic morphology of RCAs before and after carbonation. (**a**) RCA with OCS of C30; (**b**) RCA with OCS of C40; (**c**) RCA with OCS of C50; (**d**) CRCA with OCS of C30; (**e**) CRCA with OCS of C40; (**f**) CRCA with OCS of C50.

By analyzing the variation in various physical performance indexes of RCAs before and after carbonation, it was found that the macroscopic performance improvement of the dried RCA after carbonation was the best. In particular, the apparent density of the dried RCAs with C40 and C50 had the largest increase; the improvement in the water absorption and actual mass increase of dried RCAs with C50 was the most significant. The carbonation rates of the dried RCAs with C30 and C40 were the highest. Based on this, the dried RCAs with C30, C40, C50 were selected for further analysis via scanning electron microscopy before and after carbonization. The results are demonstrated in Figure 11.

Figure 11a–c show the microstructures of the RCAs, and Figure 11d–f show the microstructures of the CRCAs. By comparing and analyzing the SEM images of the microstructures of the two, it can be seen that the surface of the RCAs contained a lot of pores, with loose C-S-H gels. There were obvious microcracks between the aggregate and the old cement, and loose C-S-H gels and microcracks were obvious. After the carbonation, the carbonation product, CaCO_3_ or silica gel, compacted the microstructure of the RCAs, the number of pores and microcracks was less than that before carbonation, and the microhardness of the CRCA was significantly improved.

## 4. Discussion

Through this experimental study, it was found that the apparent density increase rate of RCAs with higher or lower OCSs after carbonation was not uniform with the change in the OCS, and the carbonation process was evidently limited by the IMC. In addition, under complete drying conditions, the water absorption rate of the RCA with a higher OCS was the largest after carbonation. After the carbonation of the untreated and completely wetted RCAs, the degree of water absorption reduction was affected by the OCS. Increasing the IMC of the RCA and the difference in the internal and external humidity gradients in the carbonation environment can effectively improve the carbonation degree of the RCA.

## 5. Conclusions and Foresight

In this paper, the influence of the OCS and IMC on the apparent density, water absorption, actual mass increase, carbonation rate and other physical properties of CRCAs was clarified by the results of accelerated carbonation experiments of RCAs. By selecting the RCA with the best carbonation modification effect to analyze the microstructure before and after carbonation, the influence of carbonation on the modification of RCA was clarified. The physical properties of RCA are greatly enhanced through carbonation, allowing it to serve as a potential substitute for NCA in the construction of reinforced concrete buildings. Additionally, it has broad applications in various infrastructure projects such as civil engineering, roads, bridges, ports, coasts, offshore, and marine engineering. The specific conclusions are as follows:

(1) Compared with that of RCA, the apparent density of CRCA increased by 0.3–3.95%. The apparent density of dried RCAs with OCSs of C40 and C50 increased the most.
(2) The water absorption of CRCA decreased by 7.98–29.56%. In particular, the dried RCAs with an OCS of C50 decreased the most.
(3) The actual mass increase of CRCA increased by 0.54–2.66%, and the actual mass increase of the dried RCAs with an OCS of C50 was the most significant.
(4) The carbonation rates of the completely dried and untreated RCAs with an OCS of C40 were the highest, with values of 29.92%.

## Figures and Tables

**Table 1:** Mixture proportions of the original concrete for RCA preparation.

Strength	W/C	Water (kg/m^3^)	Cement (kg/m^3^)	River Sand (kg/m^3^)	Crush Stone (kg/m^3^)
C30	0.6	195	325	639	1241
C40	0.5	195	390	617	1198
C50	0.4	195	488	584	1134

**Table 2:** Material factors for RCA under CO_2_-curing treatment.

Material Factors	Original Concrete Strength, OCS (MPa)	Initial Moisture Condition, IMC
MF-30-D	C30	Complete drying
MF-30-U		Untreated
MF-30-W		Complete wetting
MF-40-D	C40	Complete drying
MF-40-U		Untreated
MF-40-W		Complete wetting
MF-50-D	C50	Complete drying
MF-50-U		Untreated
MF-50-W		Complete wetting

**Table 3:** Apparent density and water absorption for NCA and RCA with different original concrete strengths.

Types of CA	OCS (MPa)	*ρ_a_* (kg/m^3^)	*W_a_* (%)
NCA	—	2710	0.33
RCA	C30	2495	5.15
	C40	2479	5.34
	C50	2450	5.50

**Table 4:** Initial moisture content for RCA with different initial moisture conditions.

OCS (MPa)	IMC	*ω_i_* (%)
C30	Complete drying	0.00
	Untreated	1.86
	Complete wetting	5.63
C40	Complete drying	0.00
	Untreated	2.66
	Complete wetting	5.88
C50	Complete drying	0.00
	Untreated	3.49
	Complete wetting	5.24

## Data Availability

Data are contained within the article.
